# Increasing Incidence of Liposarcoma: A Population-Based Study of National Surveillance Databases, 2001–2016

**DOI:** 10.3390/ijerph17082710

**Published:** 2020-04-15

**Authors:** Suzanne Bock, Douglas G. Hoffmann, Yi Jiang, Hao Chen, Dora Il’yasova

**Affiliations:** 1 Department of Population Health Sciences, School of Public Health, Georgia State University, Atlanta, GA 30303, USA; sbock5@student.gsu.edu (S.B.); dhoffmann1@student.gsu.edu (D.G.H.); 2 Department of Mathematics and Statistics, Georgia State University, Atlanta, GA 30303, USA; yjiang12@gsu.edu (Y.J.); hchen25@gsu.edu (H.C.)

**Keywords:** Liposarcoma, epidemiology, Surveillance, Epidemiology, and End Result (SEER) program, rare cancers

## Abstract

Rare cancers, affecting 1 in 5 cancer patients, disproportionally contribute to cancer mortality. This research focuses on liposarcoma, an understudied rare cancer with unknown risk factors and limited treatment options. Liposarcoma incident cases were identified from the U.S. Surveillance, Epidemiology, and End Result (SEER) program and the combined SEER-National Program of Cancer Registries (CNPCR) between 2001–2016. Incidence rates (age-adjusted and age-specific), 5-year survival, and the time trends were determined using SEER*stat software. Three-dimensional visualization of age–time curves was conducted for males and females. SEER liposarcoma cases represented ~30% (*n* = 11,162) of the nationwide pool (*N* = 37,499). Both sources of data showed males accounting for ~60% of the cases; 82%–86% cases were identified among whites. Age-adjusted incidence was greater among males vs. females and whites vs. blacks, whereas survival did not differ by sex and race. The dedifferentiated (57.2%), pleomorphic (64.1%), and retroperitoneal (63.9%) tumors had the worse survival. Nationwide, liposarcoma rates increased by 19%, with the annual percent increase (APC) of 1.43% (95% confidence interval (CI): 1.12–1.74). The APC was greater for males vs. females (1.67% vs. 0.89%) and retroperitoneal vs. extremity tumors (1.94% vs. 0.58%). Thus, incidence increased faster in the high-risk subgroup (males), and for retroperitoneal tumors, the low-survival subtype. The SEER generally over-estimated the rates and time trends compared to nationwide data but under-estimated time trends for retroperitoneal tumors. The time trends suggest an interaction between genetic and non-genetic modifiable risk factors may play a role in the etiology of this malignancy. Differences between SEER and CNCPR findings emphasize the need for nationwide cancer surveillance.

## 1. Introduction

A rare cancer, by definition, affects a small number of people. The RARECARE project dedicated to the surveillance of rare cancers in Europe defines a rare cancer as having incidence rate <6 cases per year per 100,000 population [1], while the U.S. National Cancer Institute defines it as <15 cases per year per 100,000 population [2,3]. Despite the low rate of each individual malignancy, overall rare cancers affect a significant proportion of cancer patients. In Europe, rare cancer incidence is estimated as 108 cases per 100,000, or 22% of all new annual cancer diagnoses [1]. In the U.S., 20% of all new cancer diagnoses have an incidence rate of <6 cases per 100,000 population per year [3]. Ignored by the pharmaceutical industry as well as epidemiological and clinical research, rare cancers have a considerable impact on the overall cancer outcomes. Limited treatment options, high costs of existing treatments, and frequent misdiagnosis of rare cancers burden patients, physicians, and health systems [4]. The lack of epidemiological knowledge about the risk factors associated with many rare cancers precludes the development of viable prevention, making it one of the most dramatic unresolved public health problems [1,3,5]. In comparison, well-studied cancers such as colorectal cancer, which constitutes a smaller portion of the overall cancer cases (13%), has had significant progress in cancer control and prevention [5,6]. The existing gaps in research and treatment are reflected in rare cancer survival rates. The 5-year survival for rare cancers is worse compared to common cancers: 49% vs. 63% in Europe [5]; and the U.S., 55% vs. 75% among males and 60% vs. 74% among females [4]. In light of these facts, population-based surveillance of rare cancers and their descriptive epidemiology provide the foundation for advancing etiological and clinical research with the ultimate goal of reducing the public health burden of rare cancers.

This research focuses on the under-studied rare cancer liposarcoma. Liposarcoma is a malignant mesenchymal tumor [7], accounting for almost 20% percent of adult mesenchymal tumors [8]. It can be found anywhere in the body, but most commonly in the extremities and retroperitoneum [8]. Population-based research of liposarcoma in the U.S. is limited. Previously published analyses focused on histology [9], targeted therapy [10], and the relationship between survival and tumor location or treatment [11,12]. Our descriptive analysis fills the gaps in the literature by presenting the latest and most comprehensive data available and examining liposarcoma incidence trends over time. 

In this study, we used two publicly available data sources. The Surveillance, Epidemiology, and End Result (SEER) program is the most frequently used U.S. population-based cancer incidence data. The updated dataset provides information on cancer surveillance from 18 registries across the U.S [13,14]. However, a new publicly released data source called National Program of Cancer Registries (NPCR) has recently become available. This registry covers 45 states and 96% of the U.S. population [15]. Combined, the SEER and NPCR datasets (CNPCR) provide cancer statistics covering 100% of the U.S. population [15]. While both data sources provide useful information, each has specific advantages. Although SEER represents ~27.8% of the population, it contains more detailed information on tumor characteristics and estimates of survival. CNPCR, on the other hand, has a broader representation of cancer in the U.S., but has limited information on tumor characteristics and no information on survival. Our analysis examines liposarcoma distribution by demographic and tumor characteristics as well as estimates of age-adjusted incidence rates. Age-specific incidence rates are examined in combination with the time trends of liposarcoma. Using the more detailed SEER, we delve further into liposarcoma tumor characteristics by examining the distribution of cases by grade, size, stage, and survival, providing the complete picture of liposarcoma epidemiology. 

## 2. Methods

Incident cases of adult liposarcoma for years 2001–2016 were identified using SEER 18 (November 2018) and the combined NPCR (November 2018) and SEER registry (hereafter referred as CNPCR). Cases were defined by the International Classification for Oncology, 3rd edition (ICD-0-3), codes 8850–8860 with age at diagnoses ≥18 years [13]. Annual incidence rates overall and in specific population subgroups were calculated using SEER*Stat software (version 8.3.5) [16]. The statistical significance of group comparisons was based on the calculations of 95% confidence intervals (CIs) as described by M. Fay [17]. This method considered the confidence intervals as simple functions of the inverse F-distribution with degrees of freedom calculated using directly standardized rates [17]. If the 95% CIs from different groups did not overlap, the group-difference in rates was considered to be statistically significant at the level of *p* < 0.05. The *p*-value and the statistical significance of the annual percent change (APC) in incidence was determined based on the methods described by Fay et al. [18]. All rates were expressed as cases per 100,000 person-years and age-adjusted to the 2000 U.S. standard population. 

### 2.1. Variables Available from SEER and CNCPR Datasets

In 2013, the World Health Organization (WHO) issued reclassification of soft tissue sarcomas, which redefined liposarcoma into four major histological subgroups: atypical lipomatous tumors/well-differentiated liposarcoma, dedifferentiated liposarcoma, myxoid liposarcoma, and pleomorphic liposarcoma [19,20]. Accordingly, liposarcoma histology was categorized as well-differentiated, myxoid, pleomorphic, dedifferentiated, and other (round cell, mixed, angiomyoliposarcoma, fibroblastic and not otherwise specified). Tumor site was categorized as retroperitoneal, extremities, or other. Race was categorized as white, black, or other (American Indian/Alaska Native, Asian or Pacific Islander, Hispanic). Age-specific rates were calculated for 5-year age groups starting from 20–24 (due to the low number of cases in the 18–19 age group, age-specific rates for this category was suppressed in the CNPCR data); the higher age category was 85 years and older. 

### 2.2. Variables Available from SEER Dataset 

Histologic tumor grade was considered as low (grade I) or high (grades II, III, and IV). Tumor stage was defined using SEER Historic Stage as localized (confined to the site of origin), regional/distant (direct extension and/or lymph node involvement) [21], and unknown. Given the limitation of SEER data in 2016 (retroperitoneal data was not reported), the distribution of stage and frequency of metastasis at diagnosis was described using data for the years 2001–2015. Relative 5-year survival (proportion of observed cancer survivors to expected cancer-free survivors) was calculated using SEER*stat software [16]. The 5-year relative survival estimates excluded patients with cancer that was reported through autopsy or death certificate. 

### 2.3. Time Trends

Time trends of liposarcoma age-adjusted incidence were examined using SEER*Stat. Total percent change (PC) for 2001–2016 and annual percent change (APC) with corresponding 95% CI were calculated for all cases and stratified by gender, race, and site. Visualization of trends in age-specific rates was conducted for males and females separately, with MATLAB^®^ (Matlab R2017b). The annual age-specific rates were smoothed using a moving-average filter (“smooth” function, Matlab). The rates between years were smoothly by a linear interpolation. Time and age dependencies of rates were plotted as a three-dimensional surface, where the colors correspond to the amplitude of rates.

## 3. Results

Our analyses identified 11,162 cases of liposarcoma from 2001 to 2016 in SEER, which constituted 30% of liposarcoma cases identified by CNPCR (*N* = 37,499). Distribution of liposarcoma by sex was similar in both SEER and CNCPR, with men accounting for approximately 60% of new cases. Liposarcoma was predominantly found in whites, accounting for 82%–86% of all tumors (Table 1). In SEER and CNPCR, respectively, the most common histological subtypes were well-differentiated tumors (33% and 31%), followed by other (21% and 23%), dedifferentiated (20% in both), myxoid (19% in both), and pleomorphic (7% and 8%) tumors. Most tumors were found in the extremities (39%–41%) and retroperitoneum (21%–22%), with other areas of the body accounting for 39% (Table 2). In general, both SEER and CNPCR showed similar liposarcoma age-adjusted rates, with a slight over-estimation by the SEER data. The overall age-adjusted incidence rates were estimated as 1.08 (95% CI, 1.06–1.10) and 1.01 (95% CI, 1.00–1.02) per 100,000 person-years, from SEER and CNPCR, respectively. The incidence rates were nearly twice as high for males compared to females and were greatest among whites and lowest among blacks (Table 1). 

When examined by tumor characteristics (Table 2), the highest rates were found for well-differentiated tumors (0.31–0.35 cases per 100,000 person-years). Rates of myxoid, dedifferentiated, and the category of other tumors ranged between 0.19–0.23 cases per 100,000 person-years, whereas pleomorphic tumors had the lowest incidence rate (0.08 cases per 100,000 person-years). Compared to CNPCR, SEER also slightly over-estimated the rates for tumors found in the extremities, and in sites labeled other. 

### 3.1. Age-Specific Rates from SEER and CNPCR

**Figure 1:**
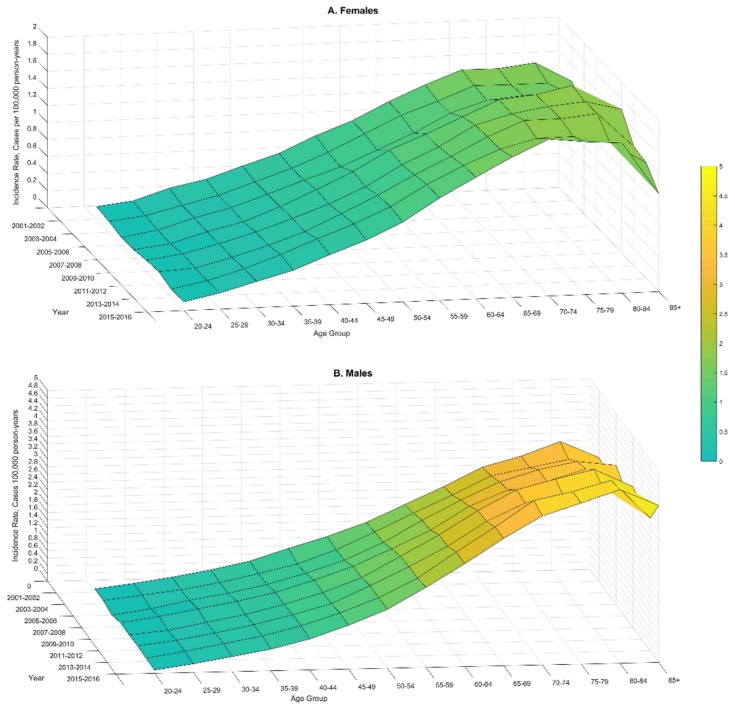
Three-dimensional visualization of age-specific incidence rates of liposarcoma among males and females during the period of 2001–2016 from the combined Surveillance, Epidemiology, and End Result and National Program of Cancer Registries (CNCPR) data.

As with many other cancers, liposarcoma incidence rates increased with age (Figure 1 and Supplemental Figure S1). The CNPCR and SEER data showed an increase in incidence among males until age 75–84 years with the peak rates of 4.36 (95% CI, 4.14–4.36) and 4.95 (95% CI, 4.58–5.35) cases per 100,000 person-years, respectively (Supplemental Figure S1). Liposarcoma rates for women reached a peak at 75–84 years with the following estimates: 1.89 (95% CI, 1.79–2.00) and 1.97 (1.74–2.22) cases per 100,000 person-years in CNPCR and SEER data, respectively (Supplemental Figure S1). 

### 3.2. Time Trends from SEER and CNPCR

The most alarming finding was the increasing liposarcoma rates from 2001 to 2016, identified by both SEER and CNPCR (Table 3, Figure 1). The annual increase of liposarcoma incidence (APC) was estimated as 1.77% (95% CI, 1.21–2.33) and 1.43% (95% CI, 1.12–1.74) by SEER and CNPCR, respectively. The overall increase estimates as percent change (PC) of liposarcoma incidence during the study period was 27.2% and 19.0 % by SEER and CNPCR, respectively. The sex-differences in time trends were under-estimated by SEER compared to CNPCR, showing almost twice greater APC in males (the demographic group that is most affected by liposarcoma; Table 3). The three-dimensional visualization of time trends (Figure 1) shows that the rise in liposarcoma incidence was more pronounced at ages 65 and older with the greatest time-differences at the peak–ages 75–84 (Figure 1). 

Stratification by race revealed the fastest growing incidence among those categorized as other, with APCs of 2.68% (95% CI, 1.69–3.68) and 3.16% (1.74, 4.60) estimated from CNPCR and SEER, respectively. Among whites, the increase in incidence nationwide was 1.6-fold greater compared to blacks (Table 3). Stratification by tumor site revealed that retroperitoneal and tumors categorized as other increased approximately by 2% a year, whereas tumors arising in extremities increased at a much slower rate with an APC of 0.58% (95% CI, 0.18–0.98), as estimated from CNPCR (Table 3). Comparing the estimates of time trends derived from SEER and CNPCR, it is clear that over-estimation of the SEER-derived trends was fairly consistent except for the analysis of retroperitoneal tumors: APC was higher as estimated by CNPCR (1.94% (95% CI, 1.40–2.49)) than by SEER (1.36% (95% CI, 0.34–2.40)). 

### 3.3. Distribution of Liposarcoma by Grade, Size, and Stage from SEER

Distribution of liposarcoma cases by tumor grade, size, and stage is presented in Supplemental Table S1. A considerable proportion of tumors (10%–19%) had missing data. The largest proportion of tumors were of low grade (46%). Examination by tumor size revealed that most tumors were ≥10 cm (58%). Examining stage distribution, we found that most tumors were diagnosed at an earlier stage (59%). Distributions of tumor grade did not differ by sex, race, and site. However, our analysis demonstrated a sharp difference in size and stage of the tumors at different sites. Retroperitoneal tumors are diagnosed as larger in size, ≥10 cm (75%), and at a more advanced (i.e., regional/distant) stage (45%). 

### 3.4. Liposarcoma Survival from SEER

Overall, 5-year relative survival did not differ by sex and race (Table 4). As expected, well-differentiated tumors predicted better survival (95.5% (95% CI, 93.6–96.9)), while pleomorphic (64.1% (95% CI, 59.1–68.7)) and dedifferentiated (57.2% (95% CI, 54.0–60.3)) indicated poorer survival. There was also a sharp difference in survival by site and grade. Tumors located on the extremities had the greatest 5-year survival rates at 89.9% (95% CI, 88.4–91.3), while retroperitoneal tumors were the deadliest (63.9% (95% CI, 61.0–66.7)). 

## 4. Discussion

This comprehensive analysis of liposarcoma included theoretically representative samples of cancer cases (SEER) and the entire U.S. population (CNPCR). Whereas generalizability of SEER data has been previously questioned [22,23], a direct comparison of cancer rates and time trends between SEER and the nationwide data (to the best of our knowledge) has not been published. Such comparison is especially meaningful for rare cancers because the nationwide data with a larger sample size provide a priori more precise estimates. Thus, one of our main objectives was to derive and compare estimates from the latest data available from SEER and CNPCR. We found reasonably similar estimates of liposarcoma incidence (for all cases) and time trends by examining both datasets, with the SEER-derived estimates being slightly greater compared to the national data. The subgroup analysis revealed that the SEER–CNCPR differences in incidence estimates were the most pronounced among males aged 60 and older, as demonstrated by the age-specific rates (Supplemental Figure S1). Such an apparent over-estimation of liposarcoma rates was not observed among females (Supplemental Figure S1). Older males account for the majority of liposarcoma tumors; thus, such sex–age differences most likely drive the observed over-estimation of liposarcoma incidence by SEER as presented in Table 1 and Table 2. 

The observed age-pattern of incidence presented a typical [24] increase followed by a plateau and a decrease at very old age (Figure 1 and Supplemental Figure S1). After age 75–80, cancer incidence is expected to decrease [25]; which may reflect age-related cellular senescence accompanied by suppression of cellular proliferation (the processes that should slow tumor growth) [25]. Alternatively, the drop in liposarcoma (or other cancers) incidence at older age reflects poorer cancer detection in the older population [24]. 

As previously published studies have focused on specific liposarcoma subtypes, there is no direct comparison for our analysis of liposarcoma demographic, histological subtypes, and tumor site distribution. Indirectly, our data can be compared to a study of liposarcoma that focuses head and neck location in relation to other sites [12]. Similar to the study by Gerry et al. [12], we found that the majority of liposarcoma cases were male (60%), white (86%)) and well-differentiated (31%). Likewise, the National Cancer Intelligence Network (NCIN) in the U.K. found twice greater liposarcoma rates among males vs. females [26].

Limited information has been published about liposarcoma time trends. The NCIN reported that liposarcoma incidence in England from 1985 to 2009 had increased [26]. These results are consistent with our findings of rising liposarcoma rates in the U.S. population (Table 3, Figure 1). The presented results indicate that the observed time trends differ by age, sex, race, and site. Alarmingly, liposarcoma rates increased in subgroups with greater risk and for the deadliest subtypes (Table 3, Figure 1). Specifically, faster increases were observed in the older population (Figure 1), among males, whites, and for the site labeled retroperitoneal (Table 3). The fact that rates are increasing faster in the subgroups with the greatest risk emphasizes the urgency to better understand the risk factors underlying such time trends.

Comparing time trends derived from SEER and CNPCR, we found the greatest difference of APC estimates for blacks; specifically, SEER over-estimated APC by almost twice compared to CNPCR (Table 3). Thus, nationwide data are particularly important when studying rare cancers among minority subgroups. When stratified by tumor location, the time trends were highly over-estimated by SEER for the tumors located in extremities, the least deadly liposarcoma (89.9% survival), whereas for the deadliest tumor (retroperitoneal (63.9% survival)) the SEER estimates were lower (Table 3). Overall, our analysis demonstrates that nationwide data are crucial in deriving a realistic picture of the trends in rare cancers. 

Consistent with published results [11], the estimates of 5-year survival did not differ between men and women (Table 4). Smith et al. showed no sex differences among well-differentiated liposarcoma in the overall and disease-specific survival [11]. However, another study that examined survival among patients with extremity myxoid liposarcomas, showed males as being a negative survival predictor [9]. For all cancer types overall, the increased cancer risk and poor survival among males has been established by population-based data [27,28,29]. Thus, our findings of similar survival among males and females must be confirmed with the more recent data when it becomes available. 

Liposarcoma histology also influences survival prognosis. Histological features such as differences in patterns of reoccurrence, metastatic risk, and grade, all influence survival [10,11,30]. Our data showed well-differentiated histology as the most common liposarcoma sub-type (33.04%) with the highest 5-year survival of 95.5% (Table 2 and Table 4). This is consistent with other liposarcoma studies [31]. In general, well-differentiated liposarcoma has a low probability of metastasis, and therefore, treatment options such as marginal excision will often yield good results [32]. However, well-differentiated tumors are also known for having a high rate of local recurrence [32]. Patients with well-differentiated tumors located in areas where excision is no longer possible, such as retroperitoneum, have lower survival (63.9%). Tumors in the retroperitoneum are often larger in size and asymptomatic until they become too big for resection [33]. Hence, retroperitoneal tumors have fewer treatment options and a poorer prognosis [34]. In agreement with published results [10,11,33], our analysis confirms that patients diagnosed with retroperitoneal tumors indeed have the lowest survival rates (Table 4). 

Mutations of recurrent well-differentiated tumors can sometimes result in a change in tumor histology. For example, dedifferentiated tumors contain patterns of both well-differentiated and other higher grade non-lipogenic elements [32]. Current estimations of more aggressive dedifferentiated nodules arising from recurrent well-differentiated tumors are around 10% [34]. Five-year survival rates for dedifferentiated tumors can be as low as 20% to 40% [32]. In our data, the 5-year survival was higher, with approximately 58% for the dedifferentiated sub-type, however, compared to the other histological types of liposarcoma where survival ranged from 96% (well-differentiated) to 64% (pleomorphic), dedifferentiated tumors are the most deadly. These statistics are not surprising given that dedifferentiated tumors are often higher grade, heterogeneous in nature, and have limited response to current therapy [32,35]. For those diagnosed with dedifferentiated tumors of the retroperitoneum, even if surgical resection is a viable treatment option, recurrence occurs in over 80% of the patients, with 30% to metastatic sites [35]. Thus, the limited treatment options for those with recurrent liposarcoma in the retroperitoneum can partially explain this poor outcome.

Given that the etiology and risk factors of liposarcoma are unknown, the increasing incidence of liposarcoma are concerning. Research of soft tissue sarcomas suggests that increasing exposure to environmental factors, such as radiation or herbicides, can provide a possible explanation for the rise in incidence rates [15,16,36]. What accounts for the detected increased incidence of liposarcoma is unclear. Our study, similar to previously published studies [12], showed greater risk among whites and males, suggesting some genetic predisposition to liposarcoma. However, such genetic factors cannot account for the recent increase in liposarcoma incidence among these subgroups. Therefore, our findings of increased incidence rates suggest an interaction between genetic predisposition and non-genetic factors, such as changes in lifestyle and environmental exposures. It has also been postulated that increasing rates of soft tissue sarcomas could be the result of better diagnostic criteria [37]. However, we would not expect better diagnostic tools, such as molecular testing, to impact well-differentiated or myxoid liposarcoma because these sub-types are easily identified by histology alone. When molecular testing is applied to undifferentiated sarcoma, it would most likely be reclassified as either pleomorphic or round cell liposarcoma. As these subtypes represent a small proportion of liposarcoma cases, we can infer that reclassification could not entirely be responsible for the observed increases in liposarcoma incidence rates. 

Our analysis has the following limitations. Our survival analysis focused only on demographic factors and tumor site and histology. As demonstrated previously [38,39,40], survival also depends on the combination of histological subtype, grading, and location. We are planning to conduct such a thorough examination of liposarcoma survival in the future. Furthermore, we did not examine survival by treatment, as this was out for the scope of this analysis. However, this important analysis should be conducted in the future. Finally, there are many individual characteristics that could be associated with the risk of liposarcoma, such as obesity and lifestyle factors. Such data are not collected by cancer registries but mostly by analytical epidemiological studies, including case-control and cohort studies. We hope that our findings will bring more attention to this rare tumor, promoting case-control and cohort studies. Whereas the U.S. is winning the war on cancer overall, not all population subgroups and cancer patients benefit from the improvements in cancer control and prevention [41]. Recent data has shown that the increase in common cancers is more pronounced in populations where the outcomes are rare. For example, cancer incidence significantly increased among younger adults in 6 out of 12 obesity-related cancers, indicating that these rare occurrences of cancer among younger individuals are happening more frequently [42]. We provide comprehensive descriptive epidemiology estimates for the under-studied rare cancer, liposarcoma. These findings will serve as a reference for current liposarcoma research; however, it is essential for future research to periodically update these estimates as data become available. Our findings emphasize the importance of nationwide data in studying rare cancers such as liposarcoma. Currently, SEER has more tumor (extent of disease such as tumor size, metastasis, etc.) and patient characteristics (place of residence at the time of diagnosis, time to death) compared to the publicly available NCPR data. In order to make progress in understanding liposarcoma etiology and track progress in treatment, future studies must be based on the nationwide data with the inclusion of as many tumor and patient characteristics as possible. Finally, we demonstrated that liposarcoma incidence is rising, suggesting the interaction between genetic and non-genetic modifiable risk factors may play a role in the etiology of this malignancy. Identification of such risk factors is necessary for the development of prevention strategies. 

## 5. Conclusions

- This analysis found that men and whites have higher risk of liposarcoma compared to other demographic subgroups.
- The liposarcoma tumors most frequently were found in the extremities and retroperitoneum.
- This population-based comprehensive analysis demonstrates increasing incident rates of liposarcoma between 2001–2016.
- The increase in incidence was mostly pronounced in high-risk demographic subgroups, suggesting an interaction between genetic and non-genetic modifiable risk factors that may play a role in liposarcoma etiology.
- Retroperitoneal tumors showed poor survival but greater increase in incidence.

The impact of the presented data is in raising awareness of the increasing liposarcoma incidence to encourage epidemiological research of its risk factors and to emphasize the importance of nationwide cancer surveillance of rare cancers.

## Figures and Tables

**Table 1:** Liposarcoma cases and age-adjusted incidence rates by demographic characteristics in the U.S. population, 2001–2016.

	SEER		CNPCR	
	Count (%)	Incidence Rate, Cases per 100,000 person-years (95% CI)	Count (%)	Incidence Rate, Cases per 100,000 person-years (95% CI)
All cases	11,162 (100)	1.08 (1.06–1.10)	37,499 (100)	1.01 (1.00–1.02)
**Sex**				
Male (ref)	6803 (60.95)	1.44 (1.41–1.48)	22,681 (60.48)	1.33 (1.31–1.35)
Female	4359 (39.05)	0.79 (0.77–0.82) *	14,818 (39.52)	0.75 (0.74–0.77)*
**Race**				
White (ref)	9198 (82.40)	1.13 (1.10–1.15)	32,186 (85.83)	1.04 (1.03–1.05)
Black	922 (8.26)	0.85 (0.7– 0.91) *	3235 (8.63)	0.81 (0.78–0.84)*
Other	1042 (9.34)	0.95 (0.89–1.01) *	2078 (5.54)	1.03 (0.99–1.08)

* Rate significantly (*p* < 0.05) different when compared to the reference category. SEER – Surveillance, Epidemiology, and End Result; CNPCR – Combined SEER-National Program of Cancer Registries; CI – Confidence Interval.

**Table 2:** Liposarcoma cases and age-adjusted incidence rates by tumor histology and site, 2001–2016.

	SEER		CNPCR	
	Count (%)	Incidence Rate, Cases per 100,000 p-years (95% CI)	Count (%)	Incidence Rate, Cases per 100,000 p-years (95% CI)
**Histological type**				
Well-differentiated (ref)	3688 (33.04)	0.35 (0.34–0.37)	11,629 (31.01)	0.31 (0.30–0.32)
Myxoid	2094 (18.76)	0.21 (0.20–0.22) *	6938 (18.50)	0.19 (0.19–0.20) *
Pleomorphic	818 (7.33)	0.08 (0.07–0.09) *	2922 (7.79)	0.08 (0.08–0.08) *
Dedifferentiated	2193 (19.65)	0.21 (0.20–0.22) *	7558 (20.16)	0.20 (0.20–0.21) *
Other ^#^	2369 (21.22)	0.23 (0.22–0.24) *	8452 (22.54)	0.23 (0.20– 0.23) *
**Tumor site**				
Extremities (ref)	4531 (40.59)	0.44 (0.43–0.45)	14,768 (39.38)	0.40 (0.40–0.41)
Retroperitoneal	2323 (20.81)	0.22 (0.21–0.23) *	8203 (21.88)	0.22 (0.21–0.22) *
Other sites	4308 (38.60)	0.42 (0.41–0.43)	14,528 (38.74)	0.39 (0.38–0.40)

* Rate significantly (*p* < 0.05) different when compared to the reference category. ^#^ Includes round cell, mixed, angiomyoliposarcoma, fibroblastic, and not otherwise specified.

**Table 3:** Time trends in liposarcoma, 2001–2016.

	SEER		CNPCR	
	Total % change	Annual % change (95% CI)	Total % change	Annual % change (95% CI)
**All cases**	27.16	1.77 (1.21–2.33)	18.95	1.43 (1.12–1.74)
**Sex**				
Males	31.53	1.87 (1.37–2.37)	21.77	1.67 (1.34–2.00)
Females	19.95	1.47 (0.68–2.27)	12.57	0.89 (0.51–1.26)
**Race/ethnicity**				
Whites	26.14	1.69 (1.01–2.37)	19.33	1.40 (1.06–1.74)
Blacks	14.01	1.62 (0.00–3.29)	4.38	0.88 (0.02–1.76)
Other	48.13	3.16 (1.74–4.60)	30.35	2.68 (1.69–3.68)
**Site**				
Extremities	2.80	1.07 (0.29–1.87)	2.89	0.58 (0.18–0.98)
Retroperitoneal	28.22	1.36 (0.34–2.40)	26.03	1.94 (1.40–2.49)
Other	64.23	2.69 (1.85–3.53)	34.41	2.02 (1.57–2.48)

**Table 4:** Five-year survival of patients with liposarcoma: SEER, 2001–2016.

	Relative Survival, % (95% CI)
**All cases**	79.8 (78.6–80.9)
**Sex**	
Male	79.0 (77.5–80.4)
Female	81.0 (79.2–82.6)
**Race**	
White	79.5 (78.2–80.8)
Black	80.9 (76.8–84.3)
Other	80.9 (77.1–84.1)
**Histological type**	
Well-differentiated	95.5 (93.6–96.9)
Myxoid	85.7 (83.5–87.7)
Pleomorphic	64.1 (59.1–68.7)
Dedifferentiated	57.2 (54.0–60.3)
Other	75.0 (72.4–77.4)
**Site**	
Retroperitoneal	63.9 (61.0 – 66.7)
Extremities	89.9 (88.4–91.3)
Other	76.7 (74.7–78.6)

## Supplementary Materials

The following are available online at https://www.mdpi.com/1660-4601/17/8/2710/s1, Figure S1: Age-specific rates of liposarcoma 2001–2016 from Surveillance, Epidemiology, and End Result (SEER) and combined SEER–National Program of Cancer Registries (CNPCR) data; Table S1: Distribution of liposarcoma tumors by grade, size, and stage: Surveillance, Epidemiology, and End Result (SEER), 2001–2016.

## Abbreviations

APC	Annual percent change
CNPCR	Combined SEER-National Program of Cancer Registries
CI	Confidence interval
NCIN	National Cancer Intelligence Network
NPCR	National Program of Cancer Registries
SEER	Surveillance, Epidemiology, and End Result
PC	Total percent change
U.K.	United Kingdom
U.S.	United States
WHO	World Health Organization
