# Supplementary material for: Increasing Incidence of Liposarcoma: A Population-Based Study of National Surveillance Databases, 2001–2016
Source: Int J Environ Res Public Health. 2020 Apr 15;17(8):2710. doi: 10.3390/ijerph17082710 (PMC7215751; doi:10.3390/ijerph17082710)

Supplemental Table 1. Distribution of liposarcoma tumors by grade, size, and stage: SEER, 2001 – 20016

	Grade, %			Size, %			Stage, %		
	Low (I)	High (II-IV)	Unknown	<10 cm	≥10cm	Unknown	Localized*	Regional/Distant*	Unknown*
**All cases**	45.9	38.7	15.4	31.4	57.5	11.1	59.3	27.5	13.2
**Sex**									
Males	44.9	40.4	14.7	32.1	56.4	11.6	59.5	26.9	13.5
Females	47.4	36.0	16.6	30.3	59.3	10.4	58.8	28.5	12.7
**Race**									
White	45.7	38.9	15.4	31.2	57.7	11.1	59.1	28.1	12.7
Black	44.1	37.1	18.8	33.6	55.4	10.8	62.7	24.4	12.9
Other	48.9	38.0	13.1	30.6	57.6	11.8	57.4	25.0	17.6
**Site**									
Retroperitoneal	44.3	40.9	14.8	16.3	74.7	9.0	42.3	44.7	13.0
Extremities	47.1	38.0	15.0	34.3	57.2	8.5	71.0	18.4	10.7
Other sites	45.5	38.2	16.3	36.3	48.6	15.1	56.1	27.9	15.9

*Due to the lack of reporting of certain cancer variables, stage was tabulated using SEER Historic Stage A (1973-2015).

Supplemental Figure 1. Age-specific rates of liposarcoma 2001-2016 from SEER and CNPCR data.

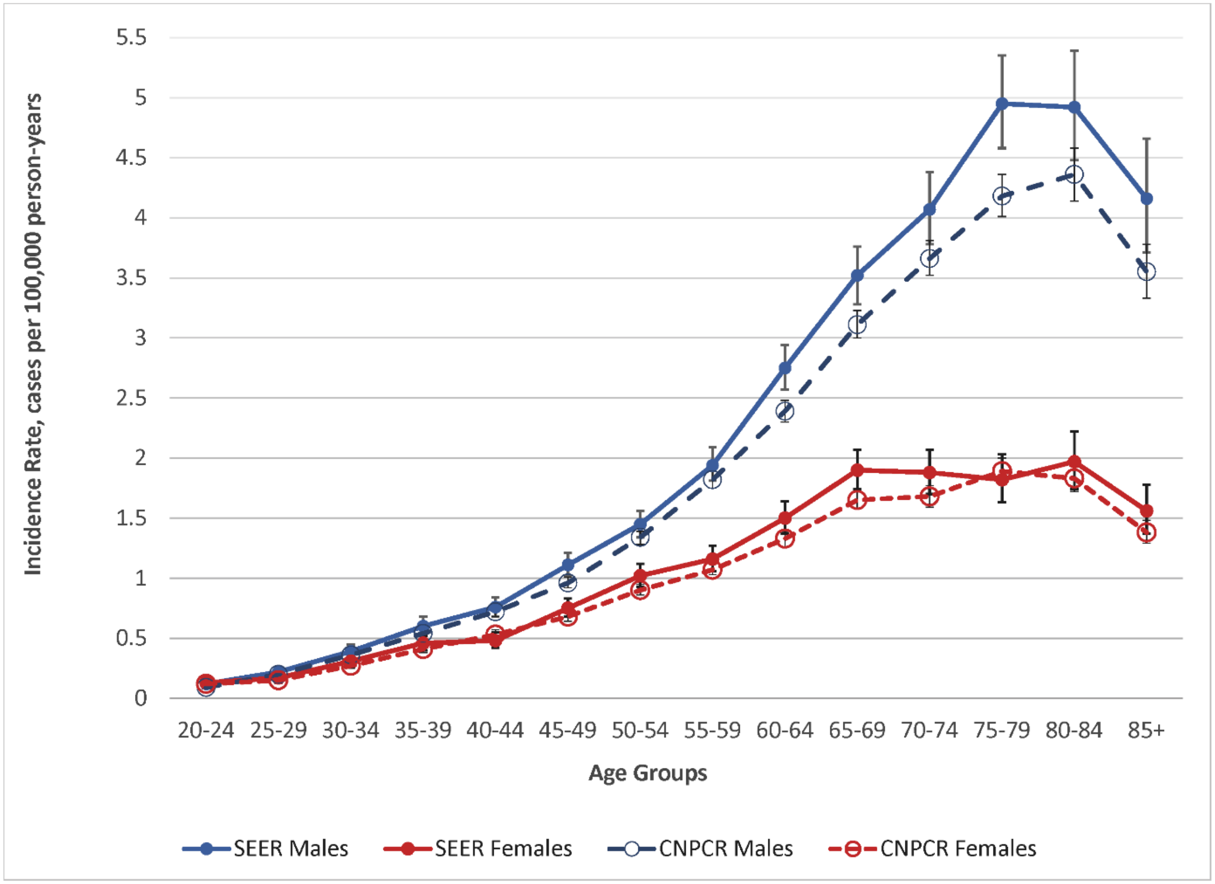

Supplement: Supplementary file 1 [file ijerph-17-02710-s001.pdf]
